# Posterior circulation stroke due to intracranial artery disease in the Chinese population

**DOI:** 10.1002/brb3.2717

**Published:** 2022-08-18

**Authors:** Changqing Zhang, Zixiao Li, Liping Liu, Yuehua Pu, Xinying Zou, Hongyi Yan, Yuesong Pan, Xingquan Zhao, Yilong Wang, Yongjun Wang

**Affiliations:** ^1^ Department of Neurology Beijing Tiantan Hospital, Capital Medical University Beijing China; ^2^ China National Clinical Research Center for Neurological Diseases Beijing China

**Keywords:** intracranial artery disease, outcome, posterior circulation stroke

## Abstract

**Background:**

Little is known about the distribution of the intracranial arteries that are responsible for noncardiogenic posterior circulation stroke (PCS) in the Chinese population. Furthermore, few studies have compared the imaging manifestations and outcomes across PCS due to the disease of different intracranial arteries. Therefore, our aim was to demonstrate the distribution of the intracranial arteries that were responsible for noncardiogenic PCS and to compare the imaging manifestations and outcome across PCS due to the disease of different intracranial arteries.

**Methods:**

We prospectively enrolled 690 patients from 22 Chinese centers with noncardiogenic PCS due to intracranial artery disease. Intracranial artery disease was classified as intracranial vertebral artery disease (IVAD) and intracranial nonvertebral artery disease (INVAD). Clinical‐radiologic patterns and outcomes were compared between IVAD and INVAD.

**Results:**

INVAD was more frequent than IVAD. Compared to the INVAD group, the IVAD group more frequently had hypertension, coronary heart disease, multiple infarcts, border zone infarcts, territorial infarcts, small cortical infarcts, multilevel infarcts, multisector infarcts, and more often had severe stenosis of the responsible artery, more often presented with decreased alertness, gaze palsy, bilateral limb weakness, ataxia, dysphagia, diplopia, vertigo, and headache. In addition, the IVAD group had a higher National Institutes of Health stroke scale score and modified Rankin Scale score at discharge and more deaths and recurrent ischemic cerebrovascular disease within 1 year of onset. Multivariable Cox regression identified IVAD as an independent predictor of recurrent ischemic cerebrovascular disease.

**Conclusions:**

PCS due to INVAD was more common in the Chinese population. However, PCS due to IVAD had more serious clinical‐radiologic patterns and worse outcomes.

## Graphical Abstract

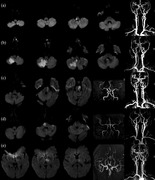

## 1 INTRODUCTION

Posterior circulation stroke (PCS) accounts for approximately 20% of ischemic stroke (IS) (Nouh et al., 2014). Noncardiogenic PCS accounts for approximately 70% of PCS, and large artery atherosclerosis (LAA) and small artery occlusion (SAO) are the most common stroke mechanisms of noncardiogenic PCS (L. Caplan, Chung, et al., 2005). Some studies report that PCS has a worse prognosis than anterior circulation stroke (Sommer et al., 2018). Vertebrobasilar artery stenosis often presents as multiple transient ischemic attacks and has a high early risk of recurrent IS (Marquardt et al., 2009). In addition, PCS caused by acute basilar artery occlusion has a very high risk of disability and death (Schonewille et al., 2009). Our previous study found that anterior circulation stroke caused by intracranial artery disease was more common than that caused by extracranial carotid artery disease in the Chinese population (Zhang et al., 2019). However, we found few studies on the distribution of the intracranial arteries that are responsible for noncardiogenic PCS in the Chinese population. Furthermore, few studies have compared the clinical‐radiologic manifestations and outcomes across PCS due to diseases of different intracranial arteries. Understanding the difference in clinical‐radiologic manifestations and prognosis between them will help to formulate corresponding treatment plans and predict the prognosis of patients. Therefore, our aim was to demonstrate the distribution of the intracranial arteries that were responsible for noncardiogenic PCS and to compare the differences in the clinical‐radiologic manifestations and outcome across PCS due to the disease of different intracranial arteries, especially the differences between those with intracranial vertebral artery disease (IVAD) and those with intracranial nonvertebral artery disease (INVAD).

## 2 METHODS

### 2.1 Subjects

**FIGURE 1:**
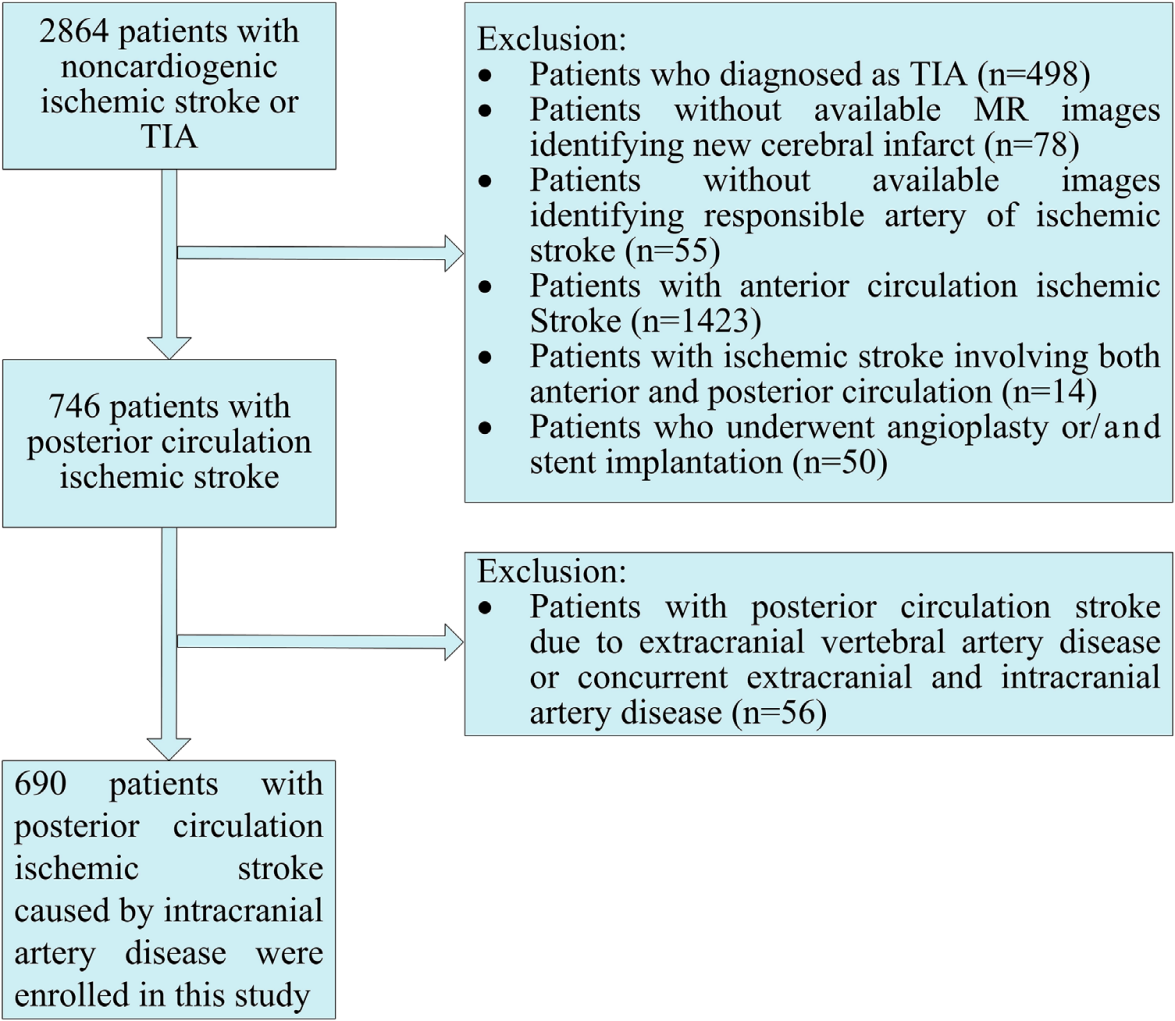
Flow chart of patient enrollment

The Chinese IntraCranial AtheroSclerosis (CICAS) Study was a prospective, multicenter, hospital‐based study. Clinical and imaging data were prospectively collected from consecutive patients with IS or transient ischemic attack (TIA) in 22 Chinese general hospitals from October 2007 to June 2009. CICAS contained data on 2864 patients with noncardiogenic ischemic cerebrovascular diseases. Those included had an onset of symptoms within 7 days and were aged between 18 and 80 years. We excluded patients with cardioembolic risk factors (atrial fibrillation, atrial flutter, valvular heart disease, bioprosthetic or mechanical heart valve replacement, myocardial infarct within the past month, sick sinus syndrome, dilated cardiomyopathy, endocarditis, etc.) or other causes of IS as well as undetermined causes. Of these, only PCS patients were enrolled, and those caused by extracranial vertebral artery (ECVA) disease or concurrent ECVA disease and intracranial artery disease were also excluded. Finally, 690 patients with noncardiogenic PCS due to intracranial artery disease were enrolled in this study. The flow chart of patient enrollment is presented in Figure 1. The CICAS protocol was approved by the ethics committee at each study center. Each participant or their legal proxies signed an informed consent form. The study conformed with the World Medical Association Declaration of Helsinki.

### 2.2 Magnetic resonance imaging analysis

All patients underwent three‐dimensional time‐of‐flight MR angiography (3D TOF MRA), axial T2‐weighted imaging, T1‐weighted imaging, fluid‐attenuated inversion recovery, and diffusion weighted imaging (DWI) sequence. All images were obtained using a 1.5‐T or 3.0‐T scanner.

**FIGURE 2:**
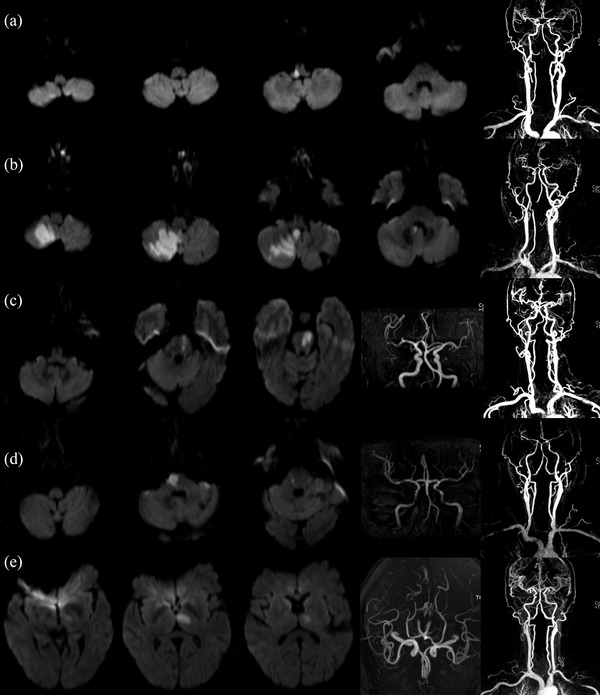
Topographical distribution of noncardiogenic posterior circulation ischemic stroke caused by intracranial artery disease. (a) Single perforating artery infarct in the right dorsolateral medulla oblongata due to severe stenosis of the right intracranial vertebral artery (ICVA). Large artery atherosclerosis (LAA) and parent artery occluding the penetrating artery were considered the etiology and stroke mechanism, respectively. (b) Multiple infarcts due to severe stenosis of the right ICVA. LAA and artery‐to‐artery embolisms were considered the etiology and the stroke mechanism, respectively. (c) Single perforating artery infarct in the left pons, with no stenosis of the basilar artery (BA). Small artery occlusion (SAO) was considered the etiology. (d) Single perforating artery infarct in the right pons, with >50% stenosis of the BA, LAA and parent artery occluding the penetrating artery, were considered the etiology and the stroke mechanism, respectively. (e) Single perforating artery infarct in the left thalamus, with no stenosis of the posterior cerebral artery. SAO was considered the etiology

New ischemic lesions were confirmed by DWI. The topographical distribution features of the acute infarcts were evaluated (Figure 2). Multiple infarcts, border zone infarcts, small cortical infarcts, and territorial infarcts were defined according to previously published methods (Zhang et al., 2019). A perforating artery infarct was defined as a single acute infarct that was located in the brainstem or thalamus and supplied by a perforating artery of the intracranial vertebral artery (ICVA), posterior inferior cerebellar artery (PICA), basilar artery (BA), anterior inferior cerebellar artery (AICA), superior cerebellar artery (SCA), posterior cerebral artery (PCA), anterior spinal artery (ASA), or posterior communicating artery (PoCA). Multisector infarcts were defined as multiple discrete infarcts supplied by two or more arteries, including the BA, bilateral VA, ASA, PICA, AICA, SCA, PoCA, and PCA. Arterial territories were determined according to Tatu and Moulin's classification of vascular anatomy (Tatu et al., 2012). PCS levels were categorized as proximal, middle, or distal (Searls et al., 2012). Infarcts located in more than one level were classified as multilevel infarcts.

The degree of intracranial artery stenosis was judged by 3D TOF MRA using the method of the Warfarin–Aspirin Symptomatic Intracranial Disease Study. The degree of extracranial artery stenosis was estimated by ultrasonography according to the published diagnostic criteria (de Bray et al., 2001) or according to the North American Symptomatic Carotid Endarterectomy Trial criteria by contrast‐enhanced magnetic resonance angiography (CEMRA) (Fox, 1993).

The artery responsible for PCS was determined according to the distribution characteristics of the acute infarcts and the results of MRA, CEMRA, or color Doppler ultrasound. The responsible intracranial artery disease was classified as IVAD and INVAD according to the location of the responsible intracranial artery. PCS caused by the disease of ICVA, or tandem lesions of ICVA and BA, were classified as IVAD, while those caused by isolated BA disease, or the disease of PICA, AICA, SCA, PCA, and PoCA, ASA were classified as INVAD.

The etiological subtypes of IS were classified according to the criteria of the Trial of ORG 10172 in Acute Stroke Treatment of the Stop Stroke Study. The stroke mechanism of LAA was further classified as follows: parent artery occluding the penetrating artery, when a single acute infarct located in the penetrating artery territory was accompanied by any degree of stenosis in the parent artery; artery‐to‐artery embolism, when single or multiple small cortical infarcts, border zone infarcts, or territory infarcts were caused by stenosis of the relevant posterior circulation arteries; and multiple mechanisms, when the two above mechanisms were present simultaneously (Gao et al., 2011).

### 2.3 Outcome evaluation

At 3, 6, and 12 months after onset, patients or their relatives were contacted by trained research personnel. The primary outcome was recurrence of IS or TIA within 1 year. All recurrent ischemic cerebrovascular events were verified at the index hospitals by the presence of new neurological deficits documented in the medical records and combining them with computed tomography or magnetic resonance imaging (MRI) findings.

### 2.4 Statistical analysis

The *χ* ^2^ test was used for comparison of categorical variables, and the Mann–Whitney *U* test was used for comparison of continuous variables having non‐normal distributions. Multivariable Cox regression was used to identify the predictors of recurrent IS or TIA within 1 year of IS. All parameters that were significant on univariate analysis with *p *< .05 or likely to have pathophysiological influence were included in the multivariable regression analysis. *p *< .05 was considered statistically significant. All analyses were performed using SAS Version 9.1.

## 3 RESULTS

The distribution of the intracranial artery responsible for PCS and the imaging features and outcomes of 690 PCS are presented in Table 1. BA was most frequently involved, followed by PCA and ICVA.

**TABLE 1:** Imaging features and outcomes of noncardiogenic posterior circulation stroke (PCS) classified by the responsible intracranial artery

	Total	ICVA	ICVA	ASA	BA	PICA	SCA	PCA
Variable	(*n *= 690)	(*n *= 113)	+ BA (*n *= 59)	(*n *= 1)	(*n *= 278)	(*n *= 2)	(*n *= 1)	(*n *= 236)
Multiple infarcts	156 (22.6)	48 (42.5)	43 (72.9)	0 (0)	14 (5.0)	1 (50.0)	1 (100)	49 (20.8)
Single perforating infarct	531 (77.0)	64 (56.6)	16 (27.1)	1 (100)	264 (95.0)	1 (50.0)	0 (0)	185 (78.4)
Border zone infarct	48 (7.0)	13 (11.5)	8 (13.6)	0 (0)	2 (0.7)	0 (0)	0 (0)	25 (10.6)
Territorial infarct	65 (9.4)	23 (20.4)	8 (13.6)	0 (0)	2 (0.7)	1 (50.0)	1 (100)	30 (12.7)
Small cortical infarct	112 (16.2)	37 (32.7)	24 (40.7)	0 (0)	7 (2.5)	1 (50.0)	0 (0)	43 (18.2)
Responsible artery stenosis ≥70% or occlusion	302 (43.8)	94 (83.2)	59 (100)	0 (0)	51 (18.3)	2 (100)	1 (100)	95 (40.3)
BA stenosis ≥70% or occlusion	110 (15.9)	0 (0)	59 (100)	0 (0)	51 (18.3)	0 (0)	0 (0)	0 (0)
BA occlusion	44 (6.4)	0 (0)	43 (72.9)	0 (0)	1 (0.4)	0 (0)	0 (0)	0 (0)
LAA Subtype	424 (61.4)	97 (85.8)	59 (100)	0 (0)	127 (45.7)	2 (100)	1 (100)	138 (58.5)
SAO subtype	266 (38.6)	16 (14.2)	0 (0)	1 (100)	151 (54.3)	0 (0)	0 (0)	98 (41.5)
Artery‐to‐artery embolism or multiple mechanisms	179 (42.2)	65 (67.0)	54 (91.5)	0 (0)	9 (7.1)	1 (50.0)	1 (100)	49 (35.5)
Level of infarct								
Proximal	63 (9.1)	54 (47.8)	6 (10.2)	1 (100)	0 (0)	2 (100)	0 (0)	0 (0)
Medial	294 (42.6)	18 (15.9)	19 (32.2)	0 (0)	257 (92.4)	0 (0)	0 (0)	0 (0)
Distal	263 (38.1)	11 (9.7)	4 (6.8)	0 (0)	11 (4.0)	0 (0)	1 (100)	236 (100)
Proximal and medial	12 (1.7)	4 (3.5)	8 (13.6)	0 (0)	0 (0)	0 (0)	0 (0)	0 (0)
Proximal and distal	8 (1.2)	8 (7.1)	0 (0)	0 (0)	0 (0)	0 (0)	0 (0)	0 (0)
Medial and distal	34 (4.9)	11 (9.7)	13 (22.0)	0 (0)	10 (3.6)	0 (0)	0 (0)	0 (0)
Proximal, medial, and distal	16 (2.3)	7 (6.2)	9 (15.3)	0 (0)	0 (0)	0 (0)	0 (0)	0 (0)
Multilevel infarcts	70 (10.1)	30 (26.5)	30 (50.8)	0 (0)	10 (3.6)	0 (0)	0 (0)	0 (0)
Multisector infarcts	67 (9.7)	28 (24.8)	31 (52.5)	0 (0)	8 (2.9)	0 (0)	0 (0)	0 (0)
Recurrent IS or TIA within 1y	36 (5.2)	9 (8.0)	12 (20.3)	0 (0)	8 (2.9)	1 (50.0)	0 (0)	6 (2.5)

Abbreviations: ASA, anterior spinal artery; BA, basilar artery; ICVA, intracranial vertebral artery; IS, ischemic stroke; LAA, large artery atherosclerosis; PCA, posterior cerebral artery; PCS, posterior circulation stroke; PICA, posterior inferior cerebellar artery; SAO, small artery occlusion; SCA, superior cerebellar artery; TIA, transient ischemic attack.

### 3.1 Clinical‐radiologic features and outcomes of the IVAD versus INVAD groups

Compared to the INVAD, the IVAD more frequently had hypertension, CHD, multiple infarcts, border zone infarcts, territorial infarcts, small cortical infarcts, multilevel infarcts, and multisector infarcts, which were more often complicated by severe stenosis or occlusion of the responsible artery. The etiology of IVAD was most often LAA, and the mechanism of IS resulting from ICVA atherosclerosis was most often artery‐to‐artery embolism or multiple mechanisms. Regarding clinical manifestations, the IVAD group more often presented with decreased alertness, gaze palsy, bilateral limb weakness, ataxia, dysphagia, diplopia, vertigo, and headache at admission, higher National Institutes of Health Stroke Scale (NIHSS) scores and modified Rankin Scale scores at discharge, a lower NIHSS score decrease at discharge, and more pneumonia and gastrointestinal bleeding during hospital stay. In addition, they also had a higher risk of recurrent ischemic cerebrovascular disease and death within 1 year of onset (Table 2).

**TABLE 2:** Clinical‐radiologic patterns and outcomes of noncardiogenic posterior circulation stroke (PCS) caused by intracranial vertebral artery disease (IVAD) versus intracranial nonvertebral artery disease (INVAD)

	INVAD	IVAD	
Variable	(*n *= 518)	(*n *= 172)	*p*‐Value
Age ≥65 years	233 (45.0)	75 (43.6)	.753
Male sex	333 (64.3)	120 (69.8)	.190
Smoking	187 (36.1)	54 (31.4)	.262
Heavy drinking	23 (4.4)	8 (4.7)	.908
Hypertension	417 (80.5)	158 (91.9)	.001
Diabetes mellitus	216 (41.7)	80 (46.5)	.269
Hyperlipidemia	398 (76.8)	131 (76.2)	.857
Coronary heart disease	38 (7.3)	23 (13.4)	.016
History of ischemic stroke	110 (21.2)	47 (27.3)	.099
Multiple infarcts	65 (12.5)	91 (52.9)	<.0001
Border zone infarct	27 (5.2)	21 (12.2)	.002
Territorial infarct	34 (6.6)	31 (18.0)	<.0001
Small cortical infarct	51 (9.8)	61 (35.5)	<.0001
Responsible artery stenosis ≥70% or occlusion	149 (28.8)	153 (89.0)	<.0001
BA stenosis ≥70% or occlusion	51 (9.8)	59 (34.3)	<.0001
LAA Subtype^a^	268 (51.7)	156 (90.7)	<.0001
Artery‐to‐artery embolism or multiple mechanisms	60 (22.4)	119 (76.3)	<.0001
Multilevel infarcts	10 (1.9)	60 (34.9)	<.0001
Multisector infarcts	8 (1.5)	59 (34.3)	<.0001
Admission signs and symptoms			
Decreased alertness	8 (1.5)	13 (7.6)	<.0001
Gaze palsy	20 (3.9)	17 (9.9)	.002
Visual field defect	28 (5.4)	13 (7.6)	.301
Facial palsy	324 (62.5)	95 (55.2)	.089
Unilateral limb weakness	294 (56.8)	72 (41.9)	.001
Bilateral limb weakness	10 (1.9)	15 (8.7)	<.0001
Ataxia	95 (18.3)	58 (33.7)	<.0001
Dysarthria	263 (50.8)	100 (58.1)	.094
Dysphagia	30 (5.8)	37 (21.5)	<.0001
Vertigo	81 (15.6)	80 (46.5)	<.0001
Diplopia	34 (6.6)	21 (12.2)	.018
Headache	24 (4.6)	15 (8.7)	.044
Repeated TIA before the stroke	14 (2.7)	4 (2.3)	1.000
Admission NIHSS, median (IQR)	4 (2,6)	4 (2,8)	.330
Admission NIHSS <4	242 (46.7)	72 (41.9)	.268
**Complications and Outcomes**			
Pneumonia	19 (3.7)	24 (14.0)	<.0001
Gastrointestinal bleeding	2 (0.4)	7 (4.1)	.001
Discharge NIHSS, median (IQR)	2 (1,3)	3 (1,5)	.001
Change between NIHSS at admission and at discharge, median (IQR)	2 (0,3)	1 (0,3)	.013
Discharge mRS, median (IQR)	1 (1,2)	2 (1,3)	.001
Recurrent IS or TIA within 1 year	15 (2.9)	21 (12.2)	<.0001
Death within 1 year	5 (1.0)	8 (4.7)	.006

Abbreviations: BA, basilar artery; IQR, interquartile range; IS, ischemic stroke; IVAD, intracranial vertebral artery disease; LAA, large artery atherosclerosis; mRS, modified Rankin Scale; NIHSS, National Institutes of Health stroke scale; TIA, transient ischemic attack.

^a^ As distinct from the small‐artery‐occlusion subtype of ischemic stroke.

### 3.2 Predictors of recurrent ischemic cerebrovascular disease within 1 year

Thirty‐six patients had recurrent IS or TIA in 1 year. Univariate analysis showed that patients with hyperlipidemia, repeated TIAs before stroke onset, multiple infarcts, multilevel infarcts, multisector infarcts, severe stenosis (≥70% or occlusion) of the responsible artery, severe BA stenosis (≥70% or occlusion), and IVAD had a higher recurrence risk (Table 3). After adjusting for age, past history, admission NIHSS, and so forth, multivariable Cox regression identified IVAD and repeated TIAs before stroke onset as predictors of recurrent ischemic cerebrovascular disease within 1 year of onset (Table 4).

**TABLE 3:** Univariate analysis for the predictors of recurrent ischemic cerebrovascular disease within 1 year

	Total	No recurrence	Recurrence	
Variables	(*n* = 690)	(*n* = 654)	(*n* = 36)	*p*‐Value
Age ≥65 years	308 (44.6)	293 (44.8)	15 (41.7)	.713
Male sex	453 (65.7)	430 (65.7)	23 (63.9)	.819
Smoking	241 (34.9)	228 (34.9)	13 (36.1)	.878
Heavy drinking	31 (4.5)	30 (4.6)	1 (2.8)	.923
Hypertension	575 (83.3)	545 (83.3)	30 (83.3)	1.000
Diabetes mellitus	296 (42.9)	279 (42.7)	17 (47.2)	.590
Hyperlipidemia	529 (76.7)	496 (75.8)	3 (91.7)	.029
Coronary heart disease	61 (8.8)	57 (8.7)	4 (11.1)	.848
History of ischemic stroke	157 (22.8)	146 (22.3)	11 (30.6)	.251
**Clinical and Imaging Features**				
Prestroke mRS, median (IQR)	0 (0,0)	0 (0,0)	0 (0,0)	.290
Admission NIHSS ≥4	376 (54.5)	357 (54.6)	19 (52.8)	.832
Discharge NIHSS ≥4	196 (28.5)	185 (28.3)	11 (32.4)	.609
Repeated TIA before the stroke	18 (2.6)	13 (2.0)	5 (13.9)	<.0001
Multiple infarcts	156 (22.6)	141 (21.6)	15 (41.7)	.005
Territorial infarct	65 (9.4)	64 (9.8)	1 (2.8)	.268
Small cortical infarct	112 (16.2)	103 (15.7)	9 (25.0)	.143
Watershed infarcts	48 (7.0)	43 (6.6)	5 (13.9)	.179
Multilevel infarcts	70 (10.1)	59 (9.0)	11 (30.6)	<.0001
Multisector infarcts	67 (9.7)	56 (8.6)	11 (30.6)	<.0001
Caused by IVAD^a^	172 (24.9)	151 (23.1)	21 (58.3)	<.0001
Responsible artery stenosis ≥70% or occlusion	302 (43.8)	276 (42.2)	26 (72.2)	<.0001
Basilar artery stenosis ≥70% or occlusion	110 (15.9)	94 (14.4)	16 (44.4)	<.0001
LAA subtype^b^	424 (61.4)	398 (60.9)	26 (72.2)	.173
**Performance measures**				
Thrombolysis at admission	20 (2.9)	18 (2.8)	2 (5.6)	.641
Antithrombotics after admission	671 (97.2)	637 (97.4)	34 (94.4)	.595
Antithrombotics at discharge	639 (92.9)	610 (93.3)	29 (85.3)	.155
Antithrombotics within 1 year	473 (69.9)	450 (69.6)	23 (76.7)	.406
Statins at discharge	481 (69.7)	459 (70.2)	22 (61.1)	.249
Statins within 1 year	257 (38.0)	243 (37.6)	14 (46.7)	.315

Abbreviations: IQR, interquartile range; IVAD, intracranial vertebral artery disease; LAA, large artery atherosclerosis; mRS, modified Rankin Scale; NIHSS, National Institutes of Health stroke scale; TIA, transient ischemic attack.

^a^ Distinct from posterior circulation stroke caused by intracranial nonvertebral artery disease.

^b^ Distinct from the small‐artery‐occlusion subtype of ischemic stroke. Data are *n* (%) unless otherwise indicated.

**TABLE 4:** Multivariable Cox regression analysis for predictors of recurrent ischemic cerebrovascular disease within 1 year

Variables	HR (95% CI)	*p*‐Value
Age ≥65 years	0.942 (0.443–2.005)	.877
Male sex	0.791 (0.348–1.798)	.576
Smoking	1.202 (0.509–2.838)	.675
Heavy drinking	0.308 (0.037–2.579)	.277
Hypertension	0.811 (0.320–2.059)	.660
Diabetes mellitus	1.081 (0.547–2.138)	.823
Hyperlipidemia	2.830 (0.847–9.457)	.091
Coronary heart disease	1.239 (0.414–3.705)	.701
History of ischemic stroke	1.434 (0.679–3.025)	.344
Repeated TIA before the stroke	9.559 (3.132–29.18)	<.0001
Admission NIHSS ≥4	0.921 (0.456–1.862)	.819
Multiple infarcts	0.577 (0.166–2.002)	.386
Multilevel infarcts	2.262 (0.621–8.247)	.216
Multisector infarcts	0.979 (0.249–3.844)	.976
Caused by IVAD^a^	3.076 (1.233–7.669)	.016
Responsible artery stenosis ≥70% or occlusion	1.525 (0.511–4.554)	.450
Basilar artery stenosis ≥70% or occlusion	2.034 (0.864–4.786)	.104

Abbreviations: CI, confidence interval; HR, hazard ratio; IVAD, intracranial vertebral artery disease; NIHSS, National Institutes of Health stroke scale; TIA, transient ischemic attack.

^a^ As distinct from posterior circulation stroke caused by intracranial nonvertebral artery disease.

## 4 DISCUSSION

Our study demonstrated the distribution of the intracranial arteries that were responsible for noncardiogenic PCS in the Chinese population. PCS due to INVAD was more common; however, PCS due to IVAD had more serious clinical‐radiologic patterns and worse outcomes.

The New England Medical Center Posterior Circulation Registry (NEMC‐PCR) found that ECVA disease was more common than ICVA, BA, and PCA disease (L. R. Caplan, 2012). However, our previous study showed that ICVA disease was more common than ECVA disease (Zhang et al., 2021). Our present study found that BA and PCA disease were more frequent than ICVA disease. Therefore, PCS due to intracranial artery disease was more common in the Chinese population. In addition, PCS due to small artery occlusion (SAO) in our study was also much higher than that in the NEMC‐PCR (38.6% vs. 14.3%) (L. Caplan, 2000). In particular, nearly half of BA and PCA diseases in our study were caused by SAO. The SAO subtype of IS belongs to the category of cerebral small vessel disease. Hypertension is the most important risk factor for cerebral small vessel disease (Filomena et al., 2015). Hypertension may increase the proportion of intracranial artery disease by causing more SAO subtypes of intracranial artery disease in our study. Our patients had a higher prevalence of hypertension than those in the NEMC‐PCR (83.3% vs. 61.4%) (L. Caplan, Wityk, et al., 2005); therefore, we speculated that the higher prevalence of hypertension may mostly account for the higher proportion of the SAO subtype of PCS and more intracranial artery disease in our study.

Our results revealed that ICVA was the main source of embolisms, similar to Caplan's findings (L. Caplan, 2000). The IVAD more frequently demonstrated an artery‐to‐artery embolic infarction pattern. Multiple artery‐to‐artery embolisms are often caused by the rupture of unstable plaques. The high recurrence risk in patients with multiple acute infarcts was most likely due to additional rupture of unstable plaque. In addition, the IVAD group more often had severe tandem stenosis (≥70% or occlusion) of the ICVA and BA. This result was consistent with Caplan's finding that ICVA occlusion was often accompanied by BA occlusion (L. R. Caplan, 2012). The sudden embolic occlusion of BA could cause severe clinical deficits due to poor compensation of the collateral flows. In addition, the reduced blood flow due to severe VA stenosis was also associated with a higher risk of recurrent PCS (Li et al., 2022). Consequently, IVAD showed more serious clinical‐radiologic patterns and a higher recurrence risk.

Dual antiplatelet therapy with aspirin and clopidogrel for 90 days proved superior to stent treatment for patients with severe symptomatic intracranial atherosclerotic stenosis (Chimowitz et al., 2011; Zaidat et al., 2015). However, some recent studies found that selected patients with severe symptomatic intracranial atherosclerotic stenosis and poor collaterals could benefit from endovascular stenting, (Ma et al., 2018; Miao et al., 2015) especially drug‐eluting stents, which could further reduce the risk of in‐stent restenosis and recurrent IS compared to bare‐metal stents (Jia et al., 2022). Therefore, IVAD patients with severe ICVA stenosis and border zone infarcts due to poor collateral compensation in the posterior circulation may be the indication for endovascular stenting. However, PCS patients with severe symptomatic intracranial atherosclerotic stenosis but no border zone infarcts may be suitable for dual antiplatelet therapy with aspirin and clopidogrel for 90 days (Chimowitz et al., 2011; Zaidat et al., 2015). The IVAD group in our study had higher proportions of severe symptomatic intracranial atherosclerotic stenosis and border zone infarcts and a higher recurrence risk than the INVAD group. Understanding the difference in clinical‐radiologic manifestations and prognosis between IVAD and INVAD will be helpful to formulate the corresponding treatment plans and to predict the prognosis of patients.

ICVA had a thicker adventitia and more vasa vasorum than BA. Vasa vasorum that grows into the plaque has immature integrity, more commonly results in the leakage of blood constituents and plaque hemorrhage, and therefore is a predictor of atherosclerotic plaque vulnerability (Yang et al., 2017). Given the critical role of vasa vasorum in the process of atherosclerosis, it may explain why the ICVA group more frequently demonstrated an artery‐to‐artery embolic infarction pattern than did the BA group.

With respect to the etiology of BA disease, SAO accounted for the largest proportion in our study. Regarding the imaging features of BA disease, a single perforating infarct was far more common than artery‐to‐artery embolism. A previous study found that vulnerable symptomatic plaque more commonly demonstrated an artery‐to‐artery embolic infarction pattern, while stable symptomatic plaque more commonly demonstrated a single perforating infarct (Kim et al., 2012). Therefore, most PCS due to BA disease in our study had no plaque or only stable plaque in the BA and thus had a lower recurrence risk.

Most single perforating infarcts located in the lateral medulla oblongata (80/81) were caused by severe stenosis or occlusion of the ICVA in our study. This was consistent with Fisher's finding and further verified that severe stenosis or occlusion of the ICVA is the most common etiology of lateral medullary infarcts (L. R. Caplan, 2012).

The etiology of most PCS due to PCA disease was LAA (138/236, 58.5%) in our study. We also found that most PCS caused by PCA disease appeared as a single perforating infarct in the thalamus, and the etiology of a single perforating infarct in the thalamus was most often SAO (98/185, 53.0%), followed by LAA (87/185, 47.0%, the stroke mechanism was parent artery occluding penetrating artery). Similar to our results, Lee et al. (2009) found that PCA territory infarcts due to SAO or parent artery occluding penetrating artery were very common in the Korean population. However, Yamamoto et al. (1999) found that most PCA territory infarcts were caused by embolisms rather than intrinsic PCA disease. This difference probably resulted from the higher prevalence of hypertension and intracranial artery disease in the Chinese population and Korean population than in the Western White population. The prevalence of hypertension in our study (83.3%) and in the Korean study (66.7%) was higher than that in the NEMC‐PCR (61.4%).

Our study has some limitations. First, patients who were clinically unstable were excluded, which may result in selection bias. Second, high‐resolution MRI was not performed, and some PCS classified as the SAO etiological subtype may have a parent artery plaque, which can be detected by high‐resolution MRI.

## CONFLICTS OF INTEREST

The authors declare no conflict of interest.

### PEER REVIEW

The peer review history for this article is available at: https://publons.com/publon/10.1002/brb3.2717.

## Data Availability

Anonymized data that support the findings of this study are available from the corresponding author upon reasonable request.
